# Early increase of cerebrospinal fluid 14-3-3ζ protein in the alzheimer's disease continuum

**DOI:** 10.3389/fnagi.2022.941927

**Published:** 2022-07-29

**Authors:** Yuanyuan Lu

**Affiliations:** Innovation Center for Neurological Disorders, Department of Neurology, Xuanwu Hospital, Capital Medical University, Beijing, China

**Keywords:** Alzheimer's disease, mild cognitive impairment, 14-3-3ζ, cerebrospinal fluid, biomarker

## Abstract

**Background:**

The earlier research has shown that the 14-3-3ζ is increased in neurofibrillary tangles (NFTs) of human Alzheimer's disease (AD) brains and stimulates the tau phosphorylation. Cerebrospinal fluid (CSF) 14-3-3ζ along the AD continuum remains to be explored.

**Methods:**

We analyzed 113 cognitive normal (CN) controls, 372 patients with mild cognitive impairment (MCI), and 225 patients with AD dementia from the Alzheimer's Disease Neuroimaging Initiative database. CSF 14-3-3ζ protein was measured by Mass Spectrometry.

**Results:**

We observed higher CSF 14-3-3ζ in the MCI group vs. the CN group and in the AD group vs. the MCI or CN group. The 14-3-3ζ was able to distinguish AD from CN and MCI. High 14-3-3ζ predicted conversion from MCI to AD. In CSF, phosphorylated tau at threonine 181 and total-tau were associated with 14-3-3ζ in MCI and AD groups, and beta-amyloid (Aβ) 42 correlated with 14-3-3ζ in the MCI group. Baseline high 14-3-3ζ was associated with cognitive decline, brain atrophy, glucose hypometabolism, and Aβ deposition in MCI and AD at baseline and follow-up.

**Conclusion:**

Our findings revealed the potential diagnostic and prognostic utility of CSF 14-3-3ζ in the AD continuum. The 14-3-3ζ could be a promising therapeutic target for the intervention of AD.

## Introduction

Alzheimer's disease (AD) is a progressive and irreversible neurodegenerative disease characterized by declined cognitive function (Winblad et al., 2016). The principal pathological hallmarks of AD are considered the extracellular beta-amyloid (Aβ) plaque deposits and neurofibrillary tangles (NFTs) in the brain (Ballard et al., 2011). NFTs disrupt neuronal function and ultimately cause neuronal death. The core cerebrospinal fluid (CSF) biomarkers Aβ42, total-tau (t-tau), and phosphorylated tau at threonine 181 (p-tau 181) have demonstrated high diagnostic validity in differentiating AD from healthy controls and other neurodegenerative dementias but with little added value in the assessment of prognosis or disease severity staging (Hampel et al., 2008; Llorens et al., 2016; Abu-Rumeileh et al., 2018). Therefore, researchers pay greatly diligent in seeking other CSF biomarkers in AD.

The 14-3-3 protein family is acidic proteins of 28–33 kDa that are abundantly expressed in the brain and particularly enriched at synapses, comprising 1% of the total soluble brain proteins (Fu et al., 2000). The seven major mammalian brain 14-3-3 isoforms are β, γ, ε, ζ, η, α (the phosphorylated forms of β), and δ (the phosphorylated forms of ζ) (Aitken et al., 1995; Chaudhri et al., 2003). The 14-3-3θ and σ are restrictedly expressed in T cells and epithelial cells, respectively (Aitken, 2006). The 14-3-3 proteins serve as central hubs for multiple biological functions and essential modulators of several vital processes in cell cycle regulation, intracellular protein transportation, synaptic plasticity, and apoptosis (Fu et al., 2000; Cau et al., 2018). Consequently, the 14-3-3 proteins are implicated in many diseases, such as metabolic diseases, neuropsychiatric disorders, and neurodegenerative diseases (Fan et al., 2019).

The 14-3-3 interplays with key proteins implicated in AD. The 14-3-3 proteins are increased in NFTs of human AD brains and stimulated the tau phosphorylation (Layfield et al., 1996). It has been reported that the 14-3-3ζ isoform is most prominently found in NFTs, indicating an isoform-specific role in AD (Umahara et al., 2004). The 14-3-3ζ binds to tau, is involved in tau phosphorylation, and regulates tau aggregation (Sluchanko et al., 2009; Qureshi et al., 2013b; Chen et al., 2019). It also interacts with δ-catenin, which was first discovered through its interaction with Presenilin-1, the molecule most frequently mutated in familial AD (He et al., 2012). In addition, the 14-3-3ζ gene modulates AD risk (Mateo et al., 2008). Few studies have investigated CSF 14-3-3ζ in AD patients so far (Park et al., 2020; Nilsson et al., 2021). In prior studies with small sample sizes, CSF 14-3-3ζ was increased in the dementia stage of AD (Park et al., 2020; Nilsson et al., 2021). However, many aspects of CSF 14-3-3ζ remain to be explored, such as the value of CSF 14-3-3ζ in disease staging across the AD continuum and its relation to cognition and neurodegeneration in longitudinal studies.

The present study was designed to explore the relationship between 14-3-3ζ in CSF and across normal cognition, mild cognitive impairment (MCI), and AD dementia in a large cohort. We tested the hypotheses that CSF 14-3-3ζ concentrations altered in the MCI stage and had diagnostic utility for AD, that 14-3-3ζ correlated with AD core biomarkers, and that high 14-3-3ζ related to cognitive impairment, brain atrophy, glucose hypometabolism, and Aβ deposition at baseline and follow-up.

## Materials and methods

### Participant characteristics

The study subjects consisted of 711 participants who had available baseline CSF 14-3-3ζ samples from the ADNI I cohort. The participants were diagnosed as cognitive normal (CN) controls (*n* = 113), MCI (*n* = 372), and AD dementia (*n* = 225) at baseline according to the established research diagnostic criteria (Aisen et al., 2010). The total follow-up period was up to 48 months. The number of subjects at different time points in the three groups is illustrated in Supplementary Table 1.

The cohort was further categorized into four different profiles (A–T–, A–T+, A+T–, and A+T+) according to the AT(N) classification proposed by the National Institute on Aging and Alzheimer's Association (NIA-AA), regardless of clinical symptoms (Jack et al., 2018). In our study, low CSF Aβ42 (<977 pg/ml) was defined as “A+”, and high CSF p-tau 181 (>27 pg/ml) as “T+” (Blennow et al., 2019).

### Genotyping analysis and CSF measurements

The APOE genotypes (gene map locus 19q13.2) were analyzed on collected blood samples for all participants. Individuals with at least one ε4 allele were defined as APOE ε4 carriers. Relative 14-3-3ζ concentrations in CSF were measured using targeted proteomics by mass spectrometry. CSF proteins from all the participants and 68 quality controls were reduced, alkylated, denatured, and enzymatically digested with lys-C and trypsin. The resulting peptides were analyzed as a single replicate over approximately 9 days using a standard flow Agilent 1,290 Infinity II liquid chromatography system coupled with Thermo Fisher Scientific TSQ Altis Triple Quadrupole mass spectrometer. Isotopically labeled peptide standards were added for relative quantification by reporting the total area ratio for the peptide (sequence VVSSIEQK) related to the 14-3-3ζ protein. The Roche Elecsys immunoassays and Elecsys cobas e 601 immunoassay analyzer systems were used for the quantification of CSF Aβ42, t-tau, and p-tau 181 as previously described (Hansson et al., 2018).

### Cognitive evaluation and neuroimaging

Global cognition was assessed by the MMSE, Alzheimer's disease Assessment Scale-cognitive subscale (ADAS-cog) 13-item score, and the Clinical Dementia Rating Scale Sum of Boxes (CDR-SB). Structural MRI brain scans were acquired from the 3.0T MR system using protocols optimized for each MR scanner. The regional volumes were quantified by the FreeSurfer pipeline. We used the data of hippocampal, entorhinal, and middle temporal volumes in this study, with adjustments for total intracranial volume (Jack et al., 2015). The 18F-fluorodeoxyglucose PET image data were acquired. Briefly, we used mean counts of the bilateral angular gyrus, bilateral posterior cingulate, and bilateral inferior temporal gyrus regions. Florbetapir was used for amyloid PET images, and each florbetapir scan was coregistered to the MRI closest in time. We used the global florbetapir SUVR from the mean counts of the frontal, anterior/posterior cingulate, lateral parietal, and lateral temporal regions. The ADNI database was used to obtain cognitive evaluation and neuroimaging for each patient at different time points.

### Statistical methods

We compared the baseline characteristics among groups by Kruskal–Wallis tests or chi-square. Associations between 14-3-3ζ and other core biomarkers were compared using Pearson's correlations. Receiver operating curve (ROC) analyses were used to assess diagnostic accuracies for each biomarker. The area under the ROC curves (AUC) with 95% confidence interval (*CI*) analyses was calculated. The associations of 14-3-3ζ with the incidence of AD were assessed by calculating hazard ratio (*HR*) with 95% *CIs* by the Cox regression analysis with adjustments for age and sex. For cognitive function measures and imaging measures, intercepts (baseline values) and slopes (rates of change) were derived using linear mixed-effects models with adjustments for age and sex. All variables were *Z*-scale transformed to ensure normality. All statistics were calculated using SPSS (v.20) and R (v.3.4.2). The statistical significance was defined as *p* < 0.05 for all analyses.

## Results

### Demographic results

The cohort included 113 CN (15.9%), 372 participants with MCI (52.4%), and 225 participants with AD dementia (31.7%). The mean age was 72.3 (±7.3) years, and education was 16.3 (±2.6) years. Among them, 340 (47.9%) were women, and 320 (47.9%) of them were APOE ε4 carriers. The baseline demographic and biomarker characteristics of the participants in each group are illustrated in Table 1. MCI group was slightly younger than the other groups (*p* < 0.001). The AD group had fewer women than the CN group (*p* < 0.01). There was no significant difference in educational levels across the three groups. There were significant differences in core CSF biomarkers, APOE ε4 status, structure imaging, and PET imaging measures across the three groups (*p* < 0.001).

**Table 1:** Study cohort characteristics at baseline.

	**CN**	**MCI**	**AD**
Age (years)	73.1(0.40)	71.2(0.39)^a^	74.3(0.78)^b^
Gender, female (%)	56.0	45.4	39.8^a^
Education (years)	16.6(0.17)	16.2(0.14)	15.7(0.25)
APOE ε4 carriers, *n* (%)	28.5	48.7	66.4^ab^
Cognitive assessment			
MMSE	29.1(0.77)	28.1(0.90)^a^	23.2(0.19)^ab^
ADAS-cog	8.9(0.29)	14.9(0.36)^a^	30.8(0.79)^ab^
CDR-SB	0.05(0.01)	1.4(0.05)^a^	4.6(0.16)^ab^
Core CSF biomarkers			
Aβ42 (pg/ml)	1033.2(30.2)	895.8(19.0)^a^	648.7(24.9)^ab^
T-tau (pg/ml)	236.4(6.24)	272.7(6.56)^a^	378.3(14.45)^ab^
P-tau181 (pg/ml)	21.7(0.64)	26.15(0.73)^a^	37.5(1.52)^ab^
Structure imaging			
Hippocampal volume	7530.9(60.82)	7056.6(61.88)^a^	5957.6(91.18)^ab^
Entorhinal volume	3852.6(42.21)	3611.2(39.88)^a^	2929.8(74.04)^ab^
Mid-temporal volume	20747.8(177.14)	20405.5(150.48)	17807.4(352.03)^ab^
PET imaging			
FDG-PET	1.32(0.07)	1.26(0.07)^a^	1.06(0.01)^ab^
Aβ-PET (AV45)	1.13(0.01)	1.22(0.01)^a^	1.41(0.02)^ab^

*Continuous variables were expressed as mean (SD). Significant differences between groups: ^a^ versus CN; ^b^ versus MCI; ^c^ versus AD*.

### Cerebrospinal fluid 14-3-3ζ concentrations in different groups

**Figure 1:**
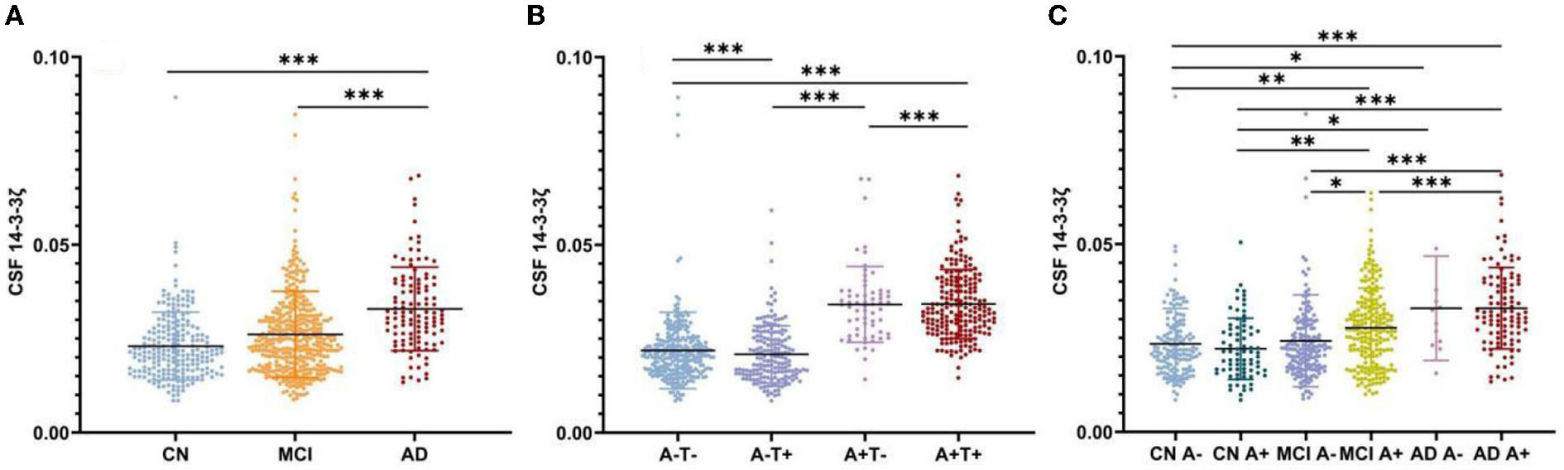
Cerebrospinal fluid (CSF) 14-3-3ζ concentrations in different groups. **(A)** CSF 14-3-3ζ concentrations in cognitive normal (CN), mild cognitive impairment (MCI), and Alzheimer's disease (AD). **(B)** CSF 14-3-3ζ concentrations by AT(N) classification. **(C)** CSF 14-3-3ζ concentrations in different clinical diagnosis and beta-amyloid (Aβ) status. *p < 0.05, **p < 0.01, ***p < 0.001.

The CSF 14-3-3ζ was elevated in patients with MCI (*p* < 0.001) and AD (*p* < 0.001) compared with CN, irrespective of Aβ status (Figure 1A). Higher 14-3-3ζ concentrations were also found in AD (*p* < 0.001) compared with MCI.

We analyzed CSF 14-3-3ζ concentrations in the four biomarker-defined subgroups (A-T-, A-T+, A+T-, and A+T+) based on AT(N) classification system (Figure 1B). We observed that CSF 14-3-3ζ was significantly higher in A+T+ than A-T- or A+T- group (*p* < 0.001). A-T+ group also had increased CSF 14-3-3ζ concentrations than A-T- or A+T- group (*p* < 0.001). Therefore, there was a large difference in 14-3-3ζ concentrations between T+ and T− individuals, but no statistical difference between A+ and A− individuals.

When considering Aβ status at different clinical stages, CSF 14-3-3ζ was found at higher concentrations in patents with MCI A+, AD A−, and AD A+ than CN A− or CN A+ individuals (Figure 1C). The 14-3-3ζ was higher in MCI A+ and AD A+ groups than MCI A− group. CSF 14-3-3ζ was also increased in AD A+ group than MCI A+ group.

### The diagnostic performance of CSF 14-3-3ζ

**Figure 2:**
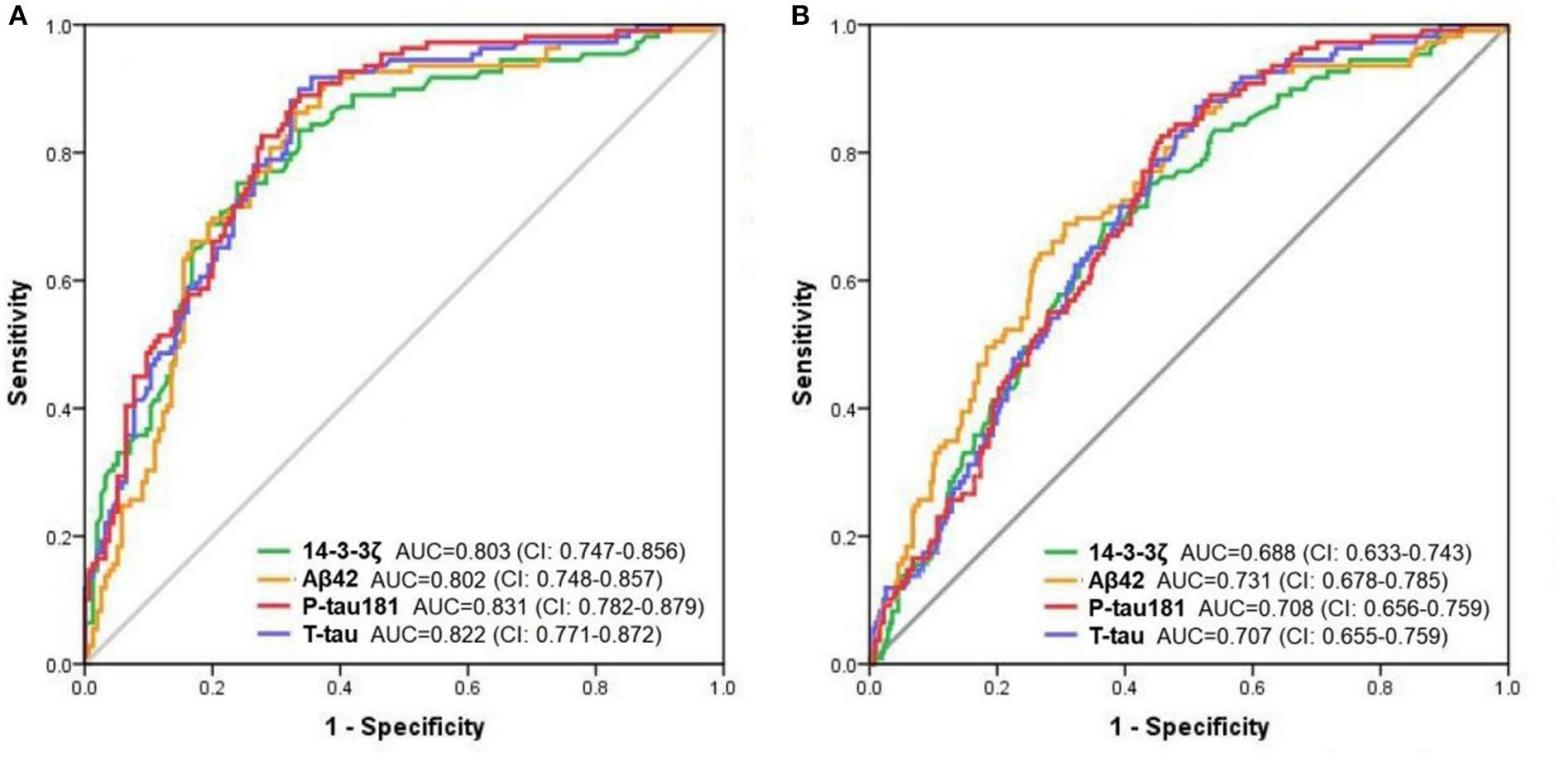
The ROC analyses. The diagnostic utility of CSF biomarkers in differentiating AD from CN **(A)** and MCI **(B)** by ROC analyses.

To test the accuracy of CSF 14-3-3ζ in distinguishing clinically diagnostic groups, we applied ROC tests to compare AD dementia patients and other groups. The diagnostic accuracy of CSF 14-3-3ζ in differentiating patients with AD from CN was as well as the core CSF markers (Figure 2A). The AUCs were 80.3% for 14-3-3ζ, 80.2% for CSF Aβ42, 82.2% for CSF t-tau, and 83.1% for CSF p-tau 181. CSF 14-3-3ζ differentiated AD from MCI (*AUC* = 68.8%) (Figure 2B). By comparison, the AUCs were 73.1% for CSF Aβ42, 70.7% for CSF t-tau, and 70.8% for CSF p-tau 181.

Furthermore, we investigated the diagnostic performance of CSF 14-3-3ζ in distinguishing biomarker-defined diagnostic groups. Comparisons of the AD A+ group and other groups revealed that 14-3-3ζ differentiated AD A+ from CN A- (AUC = 77.4%; *CI*, 71.5–83.4%) and MCI A- (*AUC* = 77.0%; *CI*, 71.2–82.9%). CSF 14-3-3ζ separated AD A+ from all A- groups (*AUC* = 72.6%; *CI*, 67.5–77.7%).

### Cerebrospinal fluid 14-3-3ζ in relation to CSF biomarkers and APOE ε4 status

**Figure 3:**
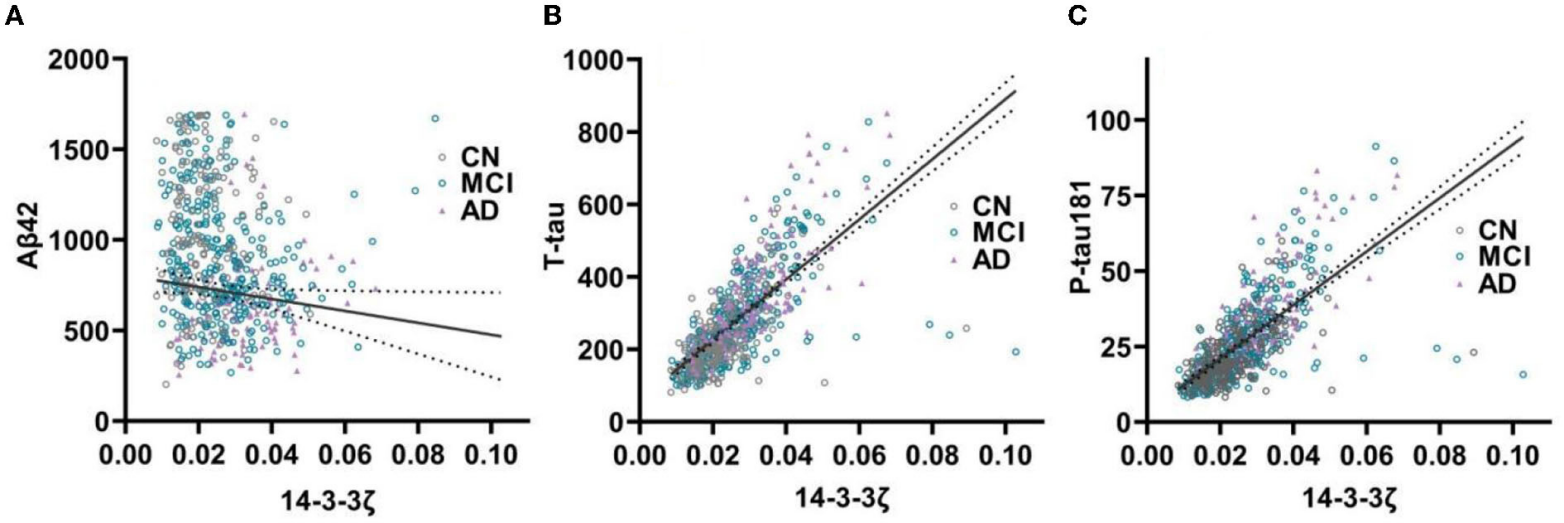
The 14-3-3ζ in relation to core biomarkers in CSF. The 14-3-3ζ related to Aβ42 **(A)**, t-tau **(B)**, and p-tau 181 **(C)**.

In the cohort, CSF 14-3-3ζ correlated with CSF Aβ42, t-tau and p-tau 181 (Figure 3). CSF t-tau had the strongest association with 14-3-3ζ, followed by CSF p-tau 181 (*r* = −0.168, *p* < 0.001 for Aβ42; *r* = 0.710, *p* < 0.001 for t-tau; and *r* = 0.695, *p* < 0.001 for p-tau 181). In each diagnostic group, Aβ42 and 14-3-3ζ were negatively correlated in MCI (*r* = −0.126, *p* = 0.026). The 14-3-3ζ and Aβ42 were not correlated in CN (*p* = 0.796) and AD individuals (*p* = 0.154). T-tau and p-tau 181 were correlated with 14-3-3ζ in CN (*r* = 0.611, *p* < 0.001 for t-tau; *r* = 0.569, *p* < 0.001 for p-tau 181), MCI (*r* = 0.685, *p* < 0.001 for t-tau; *r* = 0.672, *p* < 0.001 for p-tau 181), and AD individuals (*r* = 0.733, *p* < 0.001 for t-tau; *r* = 0.718, *p* < 0.001 for p-tau 181).

The 14-3-3ζ was elevated in APOE ε4 carries compared to none APOE ε4 carries in all individuals (*p* < 0.001). When stratified based on clinical diagnosis, 14-3-3ζ concentrations differed by APOE ε4 status in MCI patients (*p* < 0.001), but not in CN (*p* = 0.950) or AD (*p* = 0.895) individuals.

### The ability of CSF 14-3-3ζ to predict conversion from MCI to AD

**Figure 4:**
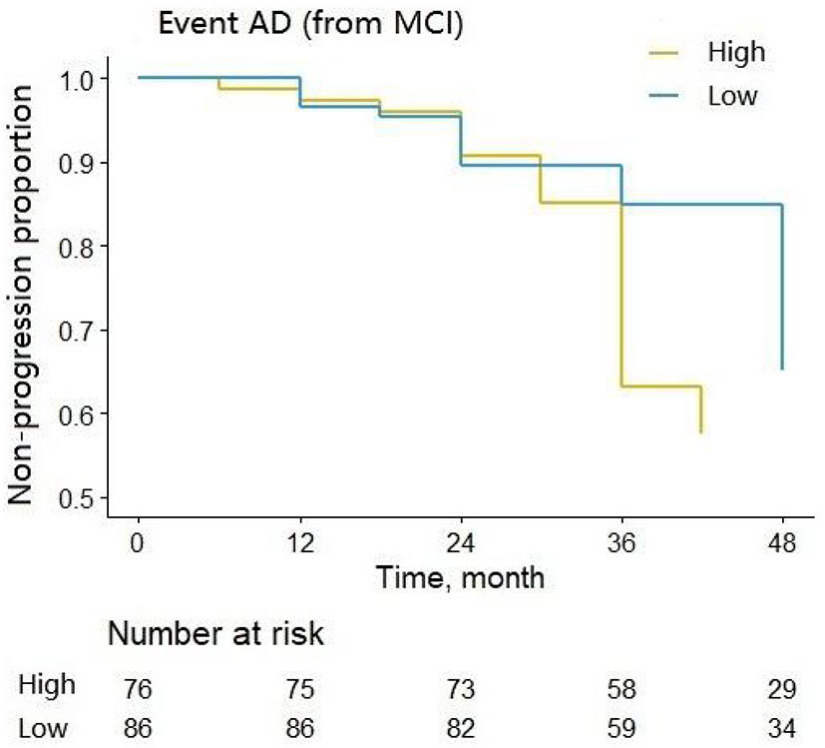
Cox regression analysis of the non-progression proportion of MCI patients with different CSF 14-3-3ζ concentrations.

We assessed the ability of CSF 14-3-3ζ to predict the likelihood of MCI to AD progression during follow-up. MCI patients were classified into two groups by the median of CSF 14-3-3ζ concentrations in the Cox regression. Individuals with high concentrations of 14-3-3ζ progressed much more rapidly to AD than individuals with low concentrations (*HR* = 2.697, *p* < 0.001) (Figure 4).

### Cerebrospinal fluid 14-3-3ζ in relation to cognitive assessments and neuroimaging

We examined whether CSF 14-3-3ζ correlated with cognitive assessments (MMSE, ADAS-Cog, and CDR-SB), structure imaging (hippocampal volume, entorhinal volume, and middle temporal volume), and PET imaging (FDG-PET and AV45-PET) (Table 2). All the variables are associated with 14-3-3ζ at baseline and follow-up. Of all the cognitive assessments, ADAS-Cog had the most significant correlation among the cognitive measures at baseline and overtime. Of all the imaging measures, AV45-PET had the most significant correlation with 14-3-3ζ at baseline, and FDG-PET had the most significant correlation with 14-3-3ζ overtime.

**Table 2:** Correlations of cerebrospinal fluid (CSF) 14-3-3ζ with cognitive scores and imaging markers.

	**Baseline**		**Follow-up**	
	**β**	***P***	**β**	***P***
Cognitive assessment				
MMSE	−0.297	<0.001	−0.248	<0.001
ADAS-cog	0.342	<0.001	0.288	<0.001
CDR-SB	0.303	<0.001	0.239	<0.001
Structure imaging				
Hippocampal volume	−0.197	<0.001	−0.189	<0.001
Entorhinal volume	−0.202	<0.001	−0.201	<0.001
Mid-temporal volume	−0.203	<0.001	−0.202	<0.001
PET imaging				
FDG-PET	−0.275	<0.001	−0.275	<0.001
Aβ-PET (AV45)	0.302	<0.001	0.237	<0.001

*Linear mixed-effects models were adjusted for age and sex*.

We also tested the influence of CSF 14-3-3ζ by diagnostic groups (Supplementary Table 2). Statistically significant interactions were found for entorhinal volume during follow-up in CN. In participants with MCI, CSF 14-3-3ζ was associated with cognitive function (MMSE, ADAS-Cog, and CDR-SB), middle temporal volume, and PET imaging (FDG-PET and AV45-PET) at baseline, and the change rates of all variables during follow-up. Among the AD group, the association remained significant for ADAS-cog at baseline and over time, as well as the change rates of MMSE, CDR-SB, entorhinal volume, mid-temporal volume, and cortical glucose metabolism during follow-up.

## Discussion

To our best knowledge, this is the first comprehensive study of the role of CSF 14-3-3ζ across normal cognition, MCI, and dementia stages of the AD continuum in a large cohort. We found that: (1) CSF 14-3-3ζ was elevated in the MCI stage and further elevated in the dementia stage. When stratified by AT(N) classification system from the NIA-AA, 14-3-3ζ concentrations were higher in A+T+ and A−T+ groups than A+T− or A−T− group. (2) 14-3-3ζ was able to distinguish AD from CN and MCI. (3) CSF p-tau 181 and t-tau were highly associated with CSF 14-3-3ζ across the AD continuum, while CSF Aβ42 was weakly correlated with CSF 14-3-3ζ in the MCI stage. (4) High concentrations of 14-3-3ζ in CSF predicted conversion from MCI to AD. (5) Baseline high CSF 14-3-3ζ was associated with cognitive decline, brain atrophy, glucose hypometabolism, and Aβ deposition in the clinical stage of the disease at baseline and follow-up. Taken together, these findings suggest that CSF 14-3-3ζ may be helpful for early diagnosis, staging of AD, and prediction of disease progression throughout the process of the AD continuum.

The 14-3-3 proteins have aroused increasing interest over the past decades and are recognized as key regulators in a wide range of neurodegenerative disorders (Burkhard et al., 2001). Despite early observations suggesting overall function similarities in 14-3-3 isoforms, later studies gradually uncovered the tissue-specific roles of individual 14-3-3 isoforms and their specific roles in neurological disorders (Muslin and Lau, 2005; Cornell and Toyo-Oka, 2017). CSF 14-3-3 protein is elevated in Creutzfeldt–Jakob disease (CJD) and has long been used clinically as a diagnostic biomarker. The previous studies found that the only isoforms of 14-3-3 that presented in the CSF of patients with CJD were β, γ, ε, and η, which could be used to differentiate CJD from other neurodegenerative diseases (Takahashi et al., 1999). In patients with Parkinson's disease (PD), the 14-3-3 expression was down-regulated in substantia nigra, and four isoforms of 14-3-3 (ε, γ, σ, and ζ) were colocalized with α-synuclein within Lewy bodies (Foote and Zhou, 2012). The 14-3-3 has been found to be involved in the neuropathology of AD. The zeta isoform of 14-3-3 has attracted increased attention as a result of its ability to interact with key proteins involved in AD (Hashiguchi et al., 2000; Foote and Zhou, 2012; He et al., 2012). Our study indicated that CSF 14-3-3ζ was already significantly elevated in the predementia stage and followed by continual increases along with disease progression in the AD continuum. We found a high diagnostic sensitivity for CSF 14-3-3ζ in differentiating patients with AD dementia from controls, which had comparable performance to the core CSF markers. CSF 14-3-3ζ was also able to distinguish biomarker-defined AD. A recent study by Nilsson et al. showed the diagnostic values of CSF 14-3-3 proteins (ζ, η, θ, and ε) in clinically diagnosed AD dementia patients. They found that all the 14-3-3 proteins had good performances in differentiating AD patients from controls (*AUC* > 0.73), with ζ showing the highest values in all proteins (*AUC* = 0.84) (Nilsson et al., 2021). In a previous study with a small number of patients, 14-3-3ζ isoform in CSF was observed to have a unique power to discriminate between AD dementia and other brain disorders, such as frontotemporal dementia, cerebrovascular disease, and Parkinson's disease (Park et al., 2020). However, the specificity of 14-3-3ζ as a diagnostic biomarker in AD still needs to be further validated in larger clinical studies in the future.

A number of studies showed that 14-3-3ζ directly interacted with tau and participated in NFTs formation (Hashiguchi et al., 2000; Umahara et al., 2004; Sluchanko et al., 2009; Qureshi et al., 2013b; Chen et al., 2019). Served as an adaptor protein, 14-3-3ζ promoted tau phosphorylation by enhancing the affinity of tau and several protein kinases, such as protein kinase A (PKA), neuronal Cdc2-like protein kinase (NCLK), and glycogen synthase kinase-3 beta (GSK3β) (Hashiguchi et al., 2000; Agarwal-Mawal et al., 2003). The 14-3-3ζ also promoted tau aggregation and caused tau fibrillization *in vitro* (Sadik et al., 2009; Qureshi et al., 2013b). Our research found that CSF 14-3-3ζ concentrations rised with tau pathology and correlated with the severity of CSF p-tau 181 and t-tau throughout the AD continuum, revealing high associations of these proteins in CSF. A study showed that there was no significant correlation between CSF 14-3-3ζ and CSF Aβ42 in AD dementia patients (Park et al., 2020). In the current study, we explored whether 14-3-3ζ was associated with Aβ as measured in the CSF and by brain imaging of Aβ deposition with Aβ-PET. We also found no correlations between 14-3-3ζ and Aβ in normal individuals or AD dementia patients. However, higher CSF 14-3-3ζ was correlated with lower CSF Aβ42, as well as increased cerebral Aβ deposition at baseline and follow-up in the MCI group. The decreased CSF Aβ42 and increased Aβ-PET uptake both reflected brain Aβ accumulation. Our findings indicated an interaction of Aβ and 14-3-3ζ at the early stage of the disease. A previous study by Nelson TJ et al. revealed a predominant protein–protein interaction of Aβ and 14-3-3 in normal rabbit brain (Nelson and Alkon, 2007). The 14-3-3 was considered an effector protein of Aβ and regulated Aβ toxicity. In another research, 14-3-3ζ was found to be significantly oxidized after injecting Aβ42 into rat brains (Boyd-Kimball et al., 2005). It is feasible that Aβ might have an effect on 14-3-3 at early stages, which created conditions for subsequent NFTs formation in AD. Further studies on the tripartite relationship among 14-3-3ζ, Aβ, and tau will be crucial to get further insights into the pathogenesis of AD.

As far as we know, the effect of 14-3-3ζ on disease progression has not been previously studied. We found that CSF 14-3-3ζ correlated with cognitive decline, brain atrophy, and brain metabolism in MCI and AD dementia stages, supporting the potential utility of CSF 14-3-3ζ as a marker of clinical progression. During follow-up, 14-3-3ζ predicted MCI conversion, and elevated 14-3-3ζ concentrations were associated with faster deterioration of cognition and neurodegeneration. Our research demonstrated that the excessive 14-3-3ζ protein might have an adverse effect during the disease progression *in vivo*. It was reported that the overexpressed 14-3-3ζ in rat hippocampal primary neurons had a detrimental effect by promoting tau phosphorylation and causing proteasomal degradation of the presynaptic protein (Qureshi et al., 2013a). Taken together, modulation of 14-3-3ζ may be a promising therapeutic approach in AD. At present, drugs targeting 14-3-3 and tau interactions are being developed (Andrei et al., 2018). Since 14-3-3 interacts with a number of downstream protein partners, challenges remain in optimizing specificity and avoiding undesirable adverse effects. It is essential to further understand the molecular basis of 14-3-3ζ dependent pathways in the pathogenesis of AD.

## Limitations

The current study has a few limitations. Firstly, our study did not include other neurologic diseases. Therefore, the diagnostic specificity of CSF 14-3-3ζ in differentiating patients with AD from non-AD neurodegenerative diseases needs to be studied. Secondly, the study lacked tau-PET data when CSF was collected. Future work investigating the association between 14-3-3ζ and the regional distribution pattern of tau pathology by tau-PET will increase our understanding of their relationships.

## Conclusions

In conclusion, our study highlighted the potential diagnostic and prognostic utility of CSF 14-3-3ζ in the AD continuum, CSF concentrations of 14-3-3ζ differentiated severity stages, and tracked disease progression in the disease. Elevated CSF 14-3-3ζ predicted more deterioration of cognitive decline, brain atrophy, glucose hypometabolism, and Aβ deposition over time. Our study provided a rationale for exploring biologics targeting 14-3-3ζ as a promising approach for AD treatment.

## Data availability statement

The raw data supporting the conclusions of this article will be made available by the authors, without undue reservation.

## Author contributions

YL wrote the manuscript, prepared figures, and reviewed the manuscript.

## Conflict of interest

The author declares that the research was conducted in the absence of any commercial or financial relationships that could be construed as a potential conflict of interest.

## Publisher's note

All claims expressed in this article are solely those of the authors and do not necessarily represent those of their affiliated organizations, or those of the publisher, the editors and the reviewers. Any product that may be evaluated in this article, or claim that may be made by its manufacturer, is not guaranteed or endorsed by the publisher.
