# Supplementary material for: Early increase of cerebrospinal fluid 14-3-3ζ protein in the alzheimer's disease continuum
Source: Front Aging Neurosci. 2022 Jul 29;14:941927. doi: 10.3389/fnagi.2022.941927 (PMC9372587; doi:10.3389/fnagi.2022.941927)
Supplement: Supplementary file 1 [file Table_1.DOCX]

Supplementary Table 1. The number of subjects included at different time points.

| **Variables** | **Time (Month)** | | | | | | |
| --- | --- | --- | --- | --- | --- | --- | --- |
|  | **Baseline** | **3** | **6** | **12** | **24** | **36** | **48** |
| **CN** |  |  |  |  |  |  |  |
| APOE ε4 | 225 |  |  |  |  |  |  |
| Aβ42 | 225 |  |  |  |  |  |  |
| T-tau | 225 |  |  |  |  |  |  |
| P-tau181 | 225 |  |  |  |  |  |  |
| 14-3-3ζ | 225 |  |  |  |  |  |  |
| MMSE | 225 |  | 211 | 155 | 188 | 34 | 112 |
| ADAS-cog | 225 |  | 207 | 153 | 187 | 32 | 112 |
| CDR-SB | 225 |  | 210 | 151 | 190 | 31 | 109 |
| Structure imaging | 202 | 148 | 119 | 142 | 162 | 10 | 91 |
| FDG-PET | 225 |  |  |  | 94 |  | 2 |
| Aβ-PET (AV45) | 222 |  |  | 1 | 161 | 2 | 92 |
| **MCI** |  |  |  |  |  |  |  |
| APOE ε4 | 372 |  |  |  |  |  |  |
| Aβ42 | 372 |  |  |  |  |  |  |
| T-tau | 372 |  |  |  |  |  |  |
| P-tau181 | 372 |  |  |  |  |  |  |
| 14-3-3ζ | 372 |  |  |  |  |  |  |
| MMSE | 372 |  | 346 | 347 | 301 | 255 | 195 |
| ADAS-cog | 371 |  | 346 | 344 | 187 | 255 | 194 |
| CDR-SB | 372 |  | 343 | 341 | 307 | 264 | 199 |
| Structure imaging | 330 | 308 | 285 | 301 | 274 | 73 | 129 |
| FDG-PET | 372 |  |  | 2 | 178 | 1 | 1 |
| Aβ-PET (AV45) | 368 |  |  | 2 |  | 2 | 133 |
| **AD** |  |  |  |  |  |  |  |
| APOE ε4 | 113 |  |  |  |  |  |  |
| Aβ42 | 113 |  |  |  |  |  |  |
| T-tau | 113 |  |  |  |  |  |  |
| P-tau181 | 113 |  |  |  |  |  |  |
| 14-3-3ζ | 113 |  |  |  |  |  |  |
| MMSE | 113 |  | 102 | 83 | 24 |  |  |
| ADAS-cog | 111 |  | 101 | 82 | 26 |  |  |
| CDR-SB | 113 |  | 102 | 85 | 26 |  | 1 |
| Structure imaging | 85 | 72 | 54 | 72 | 20 |  |  |
| FDG-PET | 112 |  |  |  | 21 |  |  |
| Aβ-PET (AV45) | 112 |  |  |  | 21 |  |  |
