# Supplementary material for: Early increase of cerebrospinal fluid 14-3-3ζ protein in the alzheimer's disease continuum
Source: Front Aging Neurosci. 2022 Jul 29;14:941927. doi: 10.3389/fnagi.2022.941927 (PMC9372587; doi:10.3389/fnagi.2022.941927)
Supplement: Supplementary file 2 [file Table_2.DOCX]

Supplementary Table 2. Correlations of CSF 14-3-3ζ with cognitive scores and imaging markers in each diagnostic group.

|  | **Baseline** | | |  | **Follow-up** | | |
| --- | --- | --- | --- | --- | --- | --- | --- |
|  | **CN** | **MCI** | **AD** |  | **CN** | **MCI** | **AD** |
| **Cognitive assessment** |  |  |  |  |  |  |  |
| MMSE | β = -0.010  *P* = 0.783 | **β = -0.115**  ***P* < 0. 001** | β = -0.098  *P* = 0.194 |  | β = 0.011  *P* = 0.476 | **β = -0.131**  ***P* < 0. 001** | **β = -0.134**  ***P* = 0.005** |
| ADAS-cog | β = 0.020  *P* = 0.585 | **β = 0.162**  ***P* = 0.000** | **β = 0.218**  ***P* = 0.009** |  | β = -0.008  *P* = 0.601 | **β = 0.183**  ***P* < 0. 001** | **β = 0.263**  ***P* < 0. 001** |
| CDR-SB | β = 0.001  *P* = 0.867 | **β = 0.062**  ***P* = 0.016** | β = 0.136  *P* = 0.154 |  | β = 0.002  *P* = 0.624 | **β = 0.080**  ***P* < 0. 001** | **β = 0.127**  ***P* < 0. 001** |
| **Structure imaging** |  |  |  |  |  |  |  |
| Hippocampal volume | β = 0.493  *P* = 0.429 | β = -0.097  *P* = 0.077 | β = -0.126  *P* = 0.866 |  | β = 0.139  *P* = 0.617 | **β = -0.118**  ***P* < 0. 001** | β = 0.058  *P* = 0.162 |
| Entorhinal volume | β = -0.566  *P* = 0.406 | β = -0.086  *P* = 0.097 | β = -0.941  *P* = 0.348 |  | **β = -0.078**  ***P* = 0.013** | **β = -0.128**  ***P* < 0. 001** | **β = -0.127**  ***P* = 0.015** |
| Mid-temporal volume | β = 0.608  *P* = 0.399 | **β = -0.143**  ***P* = 0.003** | β = -0.157  *P* = 0.157 |  | β = 0.053  *P* = 0.089 | **β = -0.171**  ***P* < 0. 001** | **β = -0.226**  ***P* < 0. 001** |
| **PET imaging** |  |  |  |  |  |  |  |
| FDG-PET | β = 0.007  *P* = 0.910 | **β = -0.148**  ***P* < 0. 001** | β = -0.016  *P* = 0.068 |  | β = 0.007  *P* = 0.883 | **β = -0.179**  ***P* < 0. 001** | **β = -0.186**  ***P* = 0.012** |
| Aβ-PET (AV45) | β = 0.091  *P* = 0.169 | **β = 0.285**  ***P* < 0. 001** | β = -0.129  *P* = 0.881 |  | β = 0.735  *P* = 0.077 | **β = 0.242**  ***P* < 0. 001** | β = 0.142  *P* = 0.859 |

Linear mixed-effects models were adjusted for age and sex. All variables were Z-scale transformed to normalize the distributions.
